# Interactive teaching of medical 3D cardiac anatomy: atrial anatomy enhanced by ECG and 3D visualization

**DOI:** 10.3389/fmed.2024.1422017

**Published:** 2024-07-04

**Authors:** Danila Potyagaylo, Peter M. van Dam, Marcin Kuniewicz, Damian Dolega-Dolegowski, Agnieszka Pregowska, Andrew Atkinson, Halina Dobrzynski, Klaudia Proniewska

**Affiliations:** ^1^Center for Digital Medicine and Robotics, Jagiellonian University Medical College, Krakow, Poland; ^2^Department of Anatomy, Jagiellonian University Medical College, Krakow, Poland; ^3^Department of Electrocardiology, Institute of Cardiology, Jagiellonian University Medical College, Krakow, Poland; ^4^Jagiellonian University Medical College, Krakow, Poland; ^5^Institute of Fundamental Technological Research, Polish Academy of Sciences, Warsaw, Poland; ^6^Division of Cardiovascular and Endocrine Sciences, University of Manchester, Manchester, United Kingdom; ^7^Department of Bioinformatics and Telemedicine, Jagiellonian University Medical College, Krakow, Poland

**Keywords:** mixed reality, CineECG, micro-CT, P wave, ECG imaging

## Abstract

The most commonly applied way of teaching students to convey the foundations of human anatomy and physiology involves textbooks and lectures. This way of transmitting knowledge causes difficulties for students, especially in the context of three-dimensional imaging of organ structures, and as a consequence translates into difficulties with imagining them. Even despite the rapid uptake of knowledge dissemination provided by online materials, including courses and webinars, there is a clear need for learning programs featuring first-hand immersive experiences tailored to suit individual study paces. In this paper, we present an approach to enhance a classical study program by combining multi-modality data and representing them in a Mixed Reality (MR)-based environment. The advantages of the proposed approach have been proven by the conducted investigation of the relationship between atrial anatomy, its electrophysiological characteristics, and resulting P wave morphology on the electrocardiogram (ECG). Another part of the paper focuses on the role of the sinoatrial node in ECG formation, while the MR-based visualization of combined micro-computed tomography (micro-CT) data with non-invasive CineECG imaging demonstrates the educational application of these advanced technologies for teaching cardiac anatomy and ECG correlations.

## 1 Introduction

It is known that teaching cardiac anatomy and electrophysiology involves conveying complex three-dimensional (3D) structures and dynamic processes, which are fundamental for understanding cardiovascular function and disease (1). Learning about the electrocardiogram (ECG) and its relation to heart anatomy is in its turn fundamental in understanding cardiac electrophysiology and diagnosing pathologies. Classical methods have traditionally focused on providing a foundational understanding through various educational tools and approaches, each with its own set of advantages and limitations.

Lectures are the oldest and an efficient way to deliver theoretical knowledge, including fundamental concepts of cardiac anatomy and the basics of electrophysiology. They are typically enhanced with visual aids, e.g. diagrams and videos, to illustrate dynamic processes. This way of learning is, however, passive in nature and may not effectively engage all students in real time. Textbooks and atlases facilitate deep understanding of complex spatial and functional concepts by providing detailed descriptions and high-quality illustrations of cardiac structures and electrical conduction pathways. They contain the basics of ECG interpretation, including detailed discussions on PQRST waves genesis, morphology, and clinical implications. These resources serve as a valuable reference for self-study and reviews, though static images are not always sufficient to fully capture the dynamic nature of cardiac electrophysiology. Concerning hands-on experience, cadaveric dissection offers an early encounter with the heart's anatomy, allowing students to appreciate the physical dimensions and spatial relationships of cardiac structures. Such an experience comes at the cost of limited functional understanding, such as electrical conduction. Furthermore, there might be scarce availability and ethical considerations in distinct countries limiting the utility of this teaching method. To overcome this limitation, physical, e.g. 3D printed, models can be manipulated to better understand the patient-specific 3D structure of the heart (2, 3). Clinical training and rotations are ECG-related hands-on practices that involve studying various ECGs to recognize normal and abnormal wave morphologies, understanding their clinical relevance, and correlating them with potential pathologies.

More recent didactic approaches include problem-based learning (PBL), simulation software tools and extended reality (XR), including virtual reality (VR) and mixed reality (MR) (4, 5). PBL promotes deeper understanding by confronting multiple clinical scenarios. It develops critical thinking and collaborative skills (6). This art of teaching requires well-designed problems, which may not cover all relevant material equally comprehensively. An analogous learning process occuring in the everyday clinical setup is through mentorship programs and case studies, where special ECG cases are discussed with experienced colleagues and presented to a broad audience in the form of scientific reports.

Simulation software for electrophysiological mapping is a very powerful tool that allows students to visualize and manipulate the electrical activity of the heart in the unprecedented ways. Moreover, the underlying excitation patterns are directly linked to the ECG enabling invasive treatment testing. A plethora of pathological conditions covering a broad range of potential scenarios with associated ECGs can be simulated for educational purposes. However, it may require a strong foundational technical knowledge to be effectively utilized (7).

VR and MR are digital animations and interactive models that an provide immersive experience for grasping truly 3D complex spatial relationships and demonstrating dynamic processes regarding cardiac electrophysiology and blood flow (8). VR simulations offer immersive experiences that can significantly enhance understanding of complex spatial relationships and functions.

Such an approach combining theoretical knowledge with practical experience and ongoing learning is crucial for understanding the complex relationship between the ECG waves and cardiac anatomy in general, and P wave on the ECG and human atrial anatomy in particular (9, 10). The heart functions fundamentally as a muscle, contracting to pump blood throughout the body. It is primarily composed of muscle cells known as cardiac myocytes. For these myocardial cells to contract, they must depolarize, a process that does not occur spontaneously, but follows a physiologically prescribed sequence triggered by natural pacemakers and realized via the conduction system. The depolarization begins in the sinoatrial node (SAN), the heart's primary pacemaker, then spreads through the contractile myocytes of the atria to reach the AV node. The AV node slowly transmits the depolarization to the His bundles, which are connected to an extensive network of small Purkinje fibers. The potential differences between adjacent myocardial cells generate currents in the extracellular space. These currents, following Ohm's law, create potentials on the body volume, detectable on its surface as the electrocardiogram (ECG). In this study we focus on the relationship between the SAN location on the highly detailed atrial anatomy, the resulting depolarization wave and the potentials measured on the body surface as the P wave.

The study of P-wave morphology is gaining interest because it can reveal the complex path of atrial depolarization and identify specific conduction issues. The characteristics of the P-wave are influenced by several factors, including (1) the starting point of the sinus rhythm, i.e. SAN, which determines the direction of depolarization in the right atrium, (2) the connection points to the left atrium, where it starts to depolarize, and (3) anatomical and tissue characteristics of the atrial chambers. In some cases, it could be challenging to ascertain if abnormalities in the P-wave morphology are due to pathological anatomical changes, e.g. atrial enlargement, or delays (or blocks) in conduction between the atria. Recent advancements in the technology for mapping the atrial endocardium connected particular P-wave shapes to specific patterns of conduction between the atria and the functionality of the primary pathways for this conduction (11). P-wave morphology analysis is important not just for identifying heart rhythm issues related to atrial conduction delays, but also for forecasting the outcomes of various cardiovascular diseases (12).

This paper aims to investigate interrelation between the atrial anatomy, P wave morphology and activation sequences, and present novel visualization options augmented by combining multi-modal data based on MR technologies, namely the application of the Microsoft HoloLens 2 device.

### 1.1 SA node location and atrial excitation

In a healthy heart, the SA node is a group of cells located in the upper part of the right atrium, near the superior vena cava (SVC) junction. Distinct approaches to analyze its size have reported substantially varying results. While electron microscopy measurements provided 13.5 ± 2.5 mm in length, 1.2 ± 0.3 mm in height, and 5.6 ± 1.4 mm in width (13), immunohistochemical methods resulted in 29.5 mm in length, 1.8 mm in height, and 6.4 mm in width (14). At the same time, a functional study analyzing atrial depolarisation in 14 patients revealed multiple patterns of impulse initiation with a pacemaker region (paranodal area) encompassing a zone as large as 75 mm × 15 mm (15). Upon SAN excitation, the area first captured by the myocardium is coined as the earliest activation site (EAS). The location of a single or multiple EASs is a subject to great inter-individual variability. In Boineau et al. (15) a typical unifocal SAN activation was observed along with unifocal extranodal impulse origin, and multicentric excitation from two to four separate atrial pacemaker sites. Furthermore, Schuessler reviewed how SAN conduction can be coupled with extranodal pacemakers and in combination with the autonomic nervous system control the pattern of atrial EAS (16).

In healthy conditions, the electrical impulse from the SAN travels through the atrial muscle in a manner facilitating coordinated and regular atrial depolarization. The fastest propagation occurs in the bundles and pectinate mucsles (PM) due to more pronounced cellular anisotropy in these regions (17). The Crista Terminals (CT), Bachmann's bundle (BB) and PM are the most prominent conductive structures, while the left atrium (LA) consists of subepicardial and subendocardial layers featuring the interatrial and septopulmonary bundles, respectively. The CT runs on the posterior wall on the right side from the SVC orifice down to the inferior vena cava orifice. The BB represents the most largest interatrial connection crossing the interatrial groove and splitting into two branches running toward atrial appendages (18). Other interatrial connections are found on the anterior and posterior sides showing a great interindividual variability.

### 1.2 P wave morphology

With the SAN in its standard (i.e. close to SVC junction) position, the P wave on the ECG usually appears as a smooth, upright deflection in the leads I, II, aVF, often biphasic in V1, and inverted in the lead aVR. Obviously, deviations in the SAN position, extent and EAS within the paranodal area can significantly impact the morphology of the P wave. Thus, an inferior SAN placement, or an inferior EAS within a normally distributed paranodal area (19), might cause a downward or negative deflection in the P wave due to the altered direction of the impulse. A posteriorly located SA node can make the P wave more pronounced in the posterior ECG leads. A shift of the SA node to the left or right may also modify P-wave morphology, affecting the interatrial connections involved and thus the direction of atrial depolarization.

Variations in P wave morphology linked to the varying SAN location can sometimes be of pathophysiological nature, e.g. due to congenital heart disease (20). Acquired changes in the interatrial muscular bridges may result in fibrotic substrate building leading to atrial fibrillation (AF) (21). Delayed interatrial conduction as measured by P wave duration has been considered as a reliable non-invasive marker associated with AF history. However, as reviewed by Platonov (11) development of paroxysmal AF is not always accompanied by underlying structural heart disease. It was further indicated that the P wave morphology depends on the three basic factors, being the SAN location defining the RA excitation pattern, the active interatrial connections and breakthrough points in the LA, and the individual atrial shape, size and structural properties. The latter factor is further affected by presence of certain pathophysiological conditions, e.g. accumulation of insoluble amyloid fibrils between cardiomyocytes promoting supraventricular arrhythmias (22). This poses the task of distinguishing morphological changes between normal anatomical variability versus those caused by pathological conditions, for instance, atrial enlargement or electrolyte imbalances.

## 2 Methods

In this study, we combine multi-modality data allowing a deeper understanding of the P wave genesis in the light of factors outlined in the previous chapter. Furthermore, it enhances visualization and thus facilitates comprehension and learning of the underlying excitation mechanisms. To achieve this two-fold goal we use micro-CT (mCT) data of the human atria, CineECG methodology and MR environment.

### 2.1 Micro-CT data

**Figure 1:**
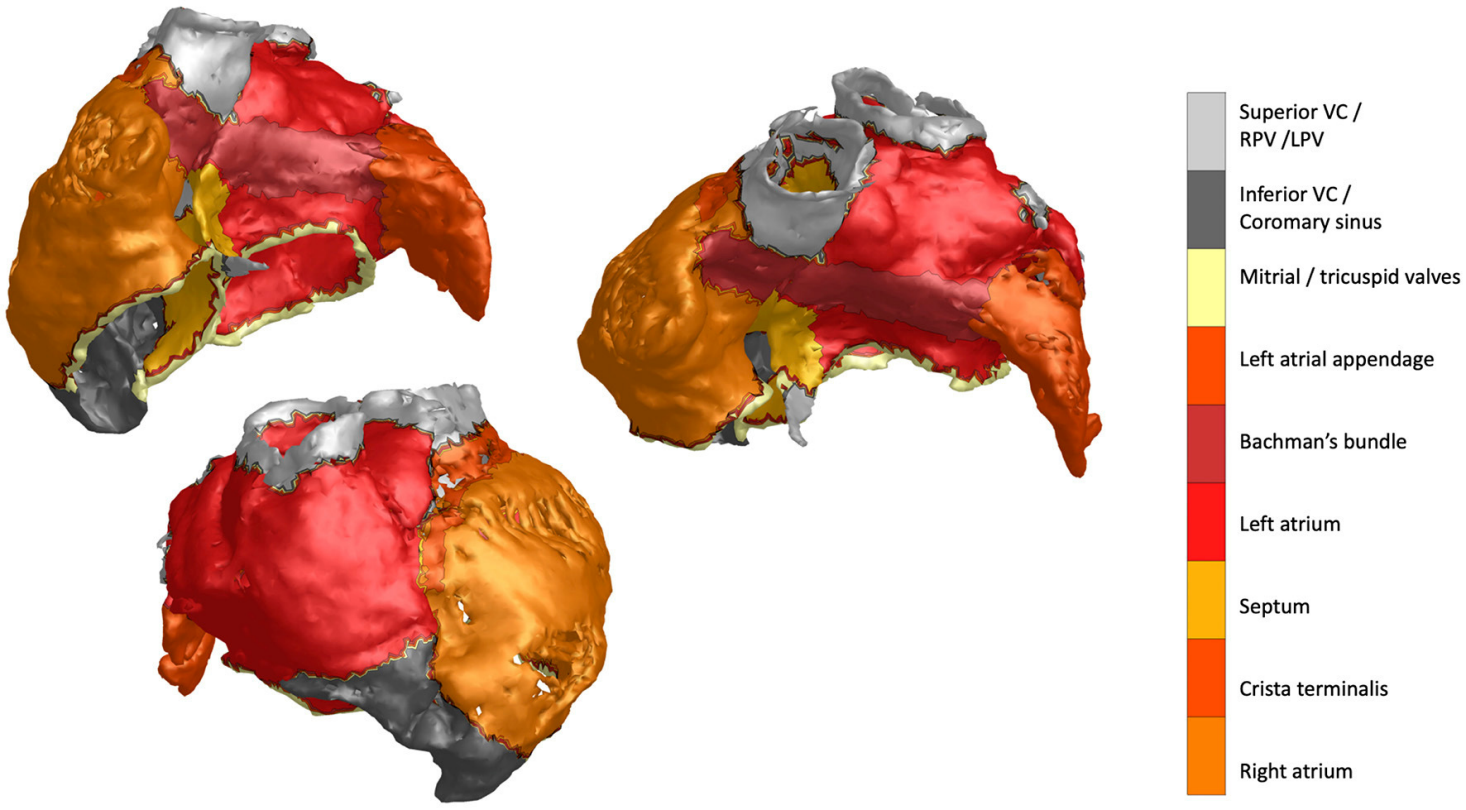
MicroCT atrial mesh data featuring distinct structures annotated by the colorbar label text.

The acquisition and processing steps for the mCT data used in this study were previously reported and described in great detail in Stephenson et al. (23). For our computation and visualization purposes we used the high-resolution triangulated meshes (498,121 nodes) as the input. The resulting atrial anatomy with color-coded distinct segmented structures is shown in Figure 1.

### 2.2 CineECG

Interpretation of 12-lead ECG is a skill requiring extensive knowledge of cardiac electrophysiology. Moreover, ECG decoding usually involves application of rule-based heuristics ignoring intrinsic anatomical relationships during wavefront propagation. With this regard, CineECG is a novel tool that allows anatomically adjusted representation of cardiac activation. CineECG approximates the path of the average electrical processes in the heart and can be best compared to estimating as the position of a moving dipole vector over time. This dipole or vector position has anatomical and electrical meaning as shown by Boonstra et al. (24). To estimate the position of the dipole vector, the vector direction needs to be estimated. This vectorcardiogram (VCG) is obtained by computing the lead vectors over time relative to the estimated vector position within the heart. For this purpose, the atrial model was used with the torso electrodes placement at the standard 12-lead positions. Based on the consectively computed VCG, the position is estimated over time by moving into the direction of the VCG with a fixed step. The obtained path represents the CineECG. It was demonstrated to correctly reflect the average location and direction of cardiac activation, recover clinically important features of the excitation spread and reliably relate an activation trajectory to the three-dimensional cardiac geometry. Beyond the proof-of-concept, this methodology has also been shown to open a novel view on the normal P waves distribution (25) and be instrumental in the 3D-based detection of an early-stage interatrial block (26).

**Figure 2:**
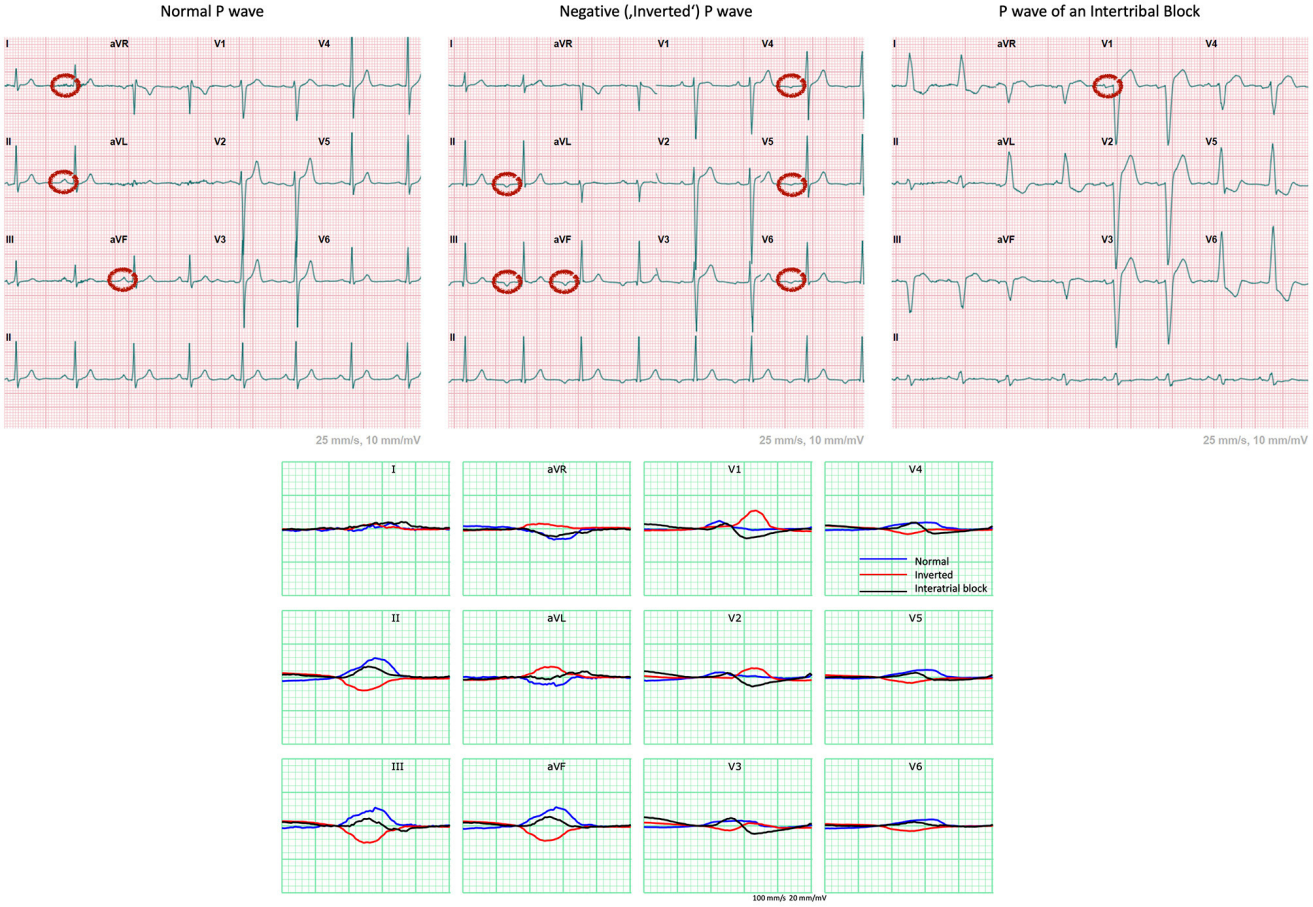
12-lead ECGs extracted from the PTB-XL ECG database showing examples of cases of a healthy normal, a negative and a P wave exhibiting an IAB. Normal P wave has positive (or upright) waves in leads I, II and aVF (11). The “inverted” P waves are negative in leads II, III, aVF, V4 to V6 and biphasic in V3. This indicates retrograde atrial conduction with the depolarization starting at the bottom of the atria, most probably originating in the atrioventricular (AV) junction. The biphasic nature of V1 indicates presence of at least partial IAB (11).

Extending on the application of this methodology to the P wave analysis, we considered the three following cases of distinct P wave morphologies highlighting the benefits of the combined representation approach to identify individualized atrial depolarization patterns related linked to a 3D anatomy. A healthy normal, a negative and a P wave morphologically indicating an interatrial block (IAB) were extracted from the PTB-XL ECG database (27). The respective ECGs are presented in Figure 2.

The mCT atrial anatomy was then fitted into a generic body model with pre-defined ECG leads location on it. Having the ECG signals and anatomical relationship between the torso and atria, we computed the mean activation trajectory through the atrial geometry. Based on this mean activation pathway we determined the EAS, corresponding to the SAN excitation breakthrough, as the surface mesh node closest to the trajectory onset. Given the excitation start, we performed the fastest route simulations to compute the activation times on the whole atrial surface given the changes in local myocardial velocities (28). For computing the initial estimates of atrial activation in the healthy and negative P waves, the conduction velocity in the BB and CT was set to be 1.5 times higher than for the normal value of 1 m/s. In the case of IAB, the BB velocity was set to 0.5 m/s. The generated excitation sequences were then further optimized to match the recorded ECGs (29).

## 3 Results

**Figure 3:**
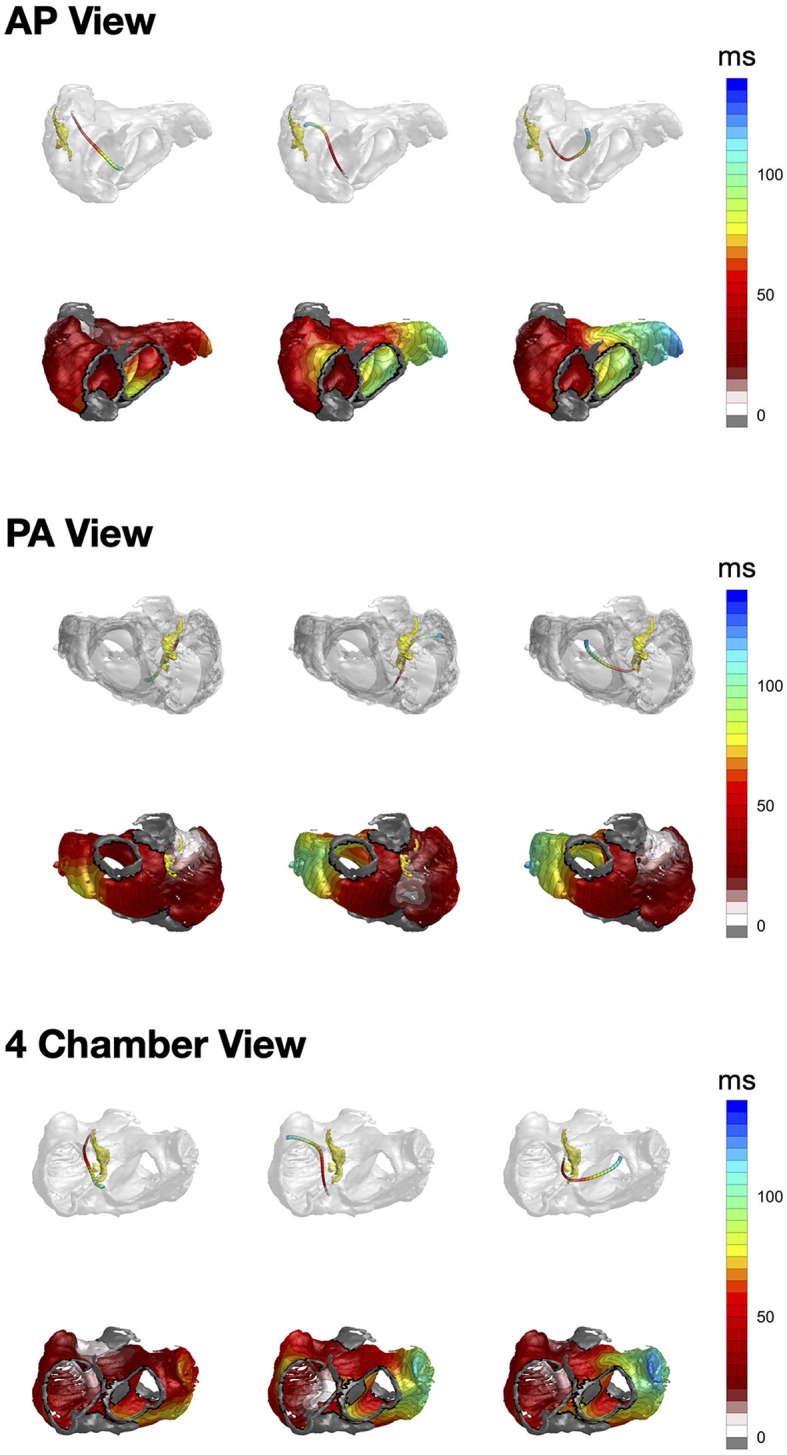
From left to right: CineECG-derived vector trajectories **(upper row)** together with the respective activation times **(lower row)** for the normal P wave, inverted P wave and interatrial block, respectively. Yellow structure in the top row is the SAN paranodal area provided by Stephenson et al. (23).

The resulting activation trajectories together with the activation times overlaid with the used atrial geometry are visualized in Figure 3. In the considered normal (positive) P wave the activation vector starts, as expected, close to the SAN area and runs in the inferior direction toward the LA. The activation times exhibit earlier depolarization on the latero-anterior RA and lateral LA walls corresponding to the faster conducting pectinate muscles and BB. The major excitation direction has a downward right-to-left pattern with the antero-lateral LA wall and IVC having the latest activation times. In contrast, activation reconstruction of the inverted P wave clearly shows the upward left-to-right propagation. The EAS is located in the inferior part of the posterior wall, and the propagation spreads to the LA and superior part of the RA. In the case of an interatrial block, the excitation pattern is markedly distinct—the trajectory leaves the paranodal area and then, instead of spreading to the LA via Bachmann's bundle and pointing downwards, goes first inferior while supposedly activating the LA via interseptal connections. The left atrial appendage exhibits expectedly the latest activation.

As Supplementary material we provide videos from the MR HoloLenses software to show user interaction while studying the presented case series.

## 4 Discussion

The presented examples indicate potential benefits of the suggested combined approach to tackle the current obstacles in the P wave analysis as well as to bridge the respective teaching gaps. Taken alone each of the methodologies, be it mCT, CineECG (or any other non-invasive ECG imaging procedure), Microsoft HoloLenses 2, can not provide a comprehensive view on the interplay between anatomy and electrophysiological function of the heart. Thus, the application of MR enhanced spatial understanding of the complex anatomical structures of the heart as well as their spatial relations. In this context, it is worth emphasizing the mutual relationship between structural cardiac changes and respective ECG waveforms. The electrical axis of the heart does not necessarily correlate to its anatomical axis reflecting possible underlying conduction disorders (30). Furthermore, it has been shown that ECG derived parameters can indicate subclinical markers of abnormal cardiac structure and function (31). Cardiomyopathy patients having very pronounced anatomical and functional alterations of the heart could benefit from careful 12-lead ECG interpretation guiding the diagnosis toward a specific disease form and providing appropriate risk stratification scores (32). Also regional disruptions of conduction properties, e.g. due to fibrotic tissue presence, result in late potentials, low voltage and fractionated local electrograms affecting the ECG genesis (33, 34).

Data obtained with the dedicated mCT protocol allow a thorough examination and delineation of all anatomical structures and substrates relevant for atrial electrical excitation, including the SAN, fast conducting fibers (CT, BB, intercaval, septopulmunory and septoatrial bundles, pectinate muscles). While mCT offers an unparalleled high resolution compared to conventional clinical CT scanners, it comes at cost of much high radiation power and exposure times. As dose and motion artifacts are less of a concern in cadaveric tissues, the mCT data can be obtained and processed only post-mortem. Therefore we needed to augment the geometry with ECG recordings from other individuals. The PTB-XL represents an excellent well-documented source of ECG data featuring a variety of normal and abnormal cases. For the proof-of-concept and illustration purposes we picked a healthy normal, an inverted P wave and a case with an interatrial block. As CineECG estimates excitation patterns based on the anatomical relationships between the heart, torso and 12-lead ECG electrodes, we fitted the mCT atria into a generic body with pre-specified lead locations. In principle, this procedure can be applied to any generic torso-heart pair, however, overlaying the resulting excitation pattern could be of greater clinical and teaching significance. Highlighting areas with slow or blocked conduction and breakthrough points in the RA and LA matched with the anatomical mCT template can serve as a valuable tool for 3D visualization facilitating ECG interpretation and diagnosis. Even further enhancement in data representation and show-casing is provided by the Microsoft HoloLens 2 technology allowing an immersive experience and thus amended understanding of the relationship between cardiac anatomy and electrical activity. It is worth noting that an MR-based environment by combining virtual content with the real world, not only makes it more attractive but also increases the level of student engagement (35). It is also an effective environment that supports collaborative learning (36).

In research, such a combined approach can aid in studying the impact of anatomical variations. For the same ECG excitation pathways might be distinct, and performing this analysis would provide the spectrum of possible conduction disorders. Detailed mCT data together with the HoloLenses can also be augmented by a cardiac simulation tool, e.g. Plank et al. (7). Then all anatomical structures would be incorporated into the modeling procedure and different pathological scenarios, i.e. excitation sequences together with the respective ECGs, can be simulated and visualized for educational purposes. In a clinical setting, integrating patient-specific ECG data with the template mCT anatomical data can assist in planning and guiding cardiac procedures, such as ablations, by providing a detailed map of the cardiac structures and electrical activation patterns.

## 5 Limitations

The needed CT resolution can not be currently implemented in the clinical patient-screening settings, making the combination of patient-specific ECG with a template mCT data necessary for clinical applications of the combined approach. However, we believe it to be a great fit for educational purposes merging anatomical and electrophysiological data in a user-friendly visualization environment. The CineECG tool aims to provide a novel view of cardiac excitation facilitating ECG students and practitioners in patients' screening and diagnostic. It does not deliver an activation map competing in accuracy with a result of the invasive mapping study. Nevertheless, it was shown to provide additional information at the spatial level that can be related to the atrial conduction system (26). Finally, the Hololenses as a tool requires specialized software and hardware incurring the purchase cost. Thus, the implementation of MR-based imaging technology in educational practice due to high cost, which is particularly important in low-income countries may be troublesome (37). For many educational units, such an expense may turn out to be too high. This may have a potential influence on deepening already existing inequalities in education. Furthermore, some knowledge in both cardiac imaging and electrophysiology constitute the pre-requisites for the HoloLenses to be run effectively. MR-based systems may also be quite complicated in configuration and maintenance. Another limitation is connected with the introduction of some kind of dependence of the learning process on technology. In this case, technical failures, software glitches, or hardware failures may disrupt this process. Additionally, prolonged use of MR technology may cause physical discomfort or fatigue, including headaches and motion sickness (38).

## 6 Conclusions

To summarize, the combination of mCT data with ECG analysis for visualization purposes is a powerful approach that can enhance our understanding of atrial electrophysiology, aid in medical education, and have clinical applications in diagnosing and treating cardiac conditions. The present cases will be made publicly available in the HoloLenses-compatible format to bring more awareness to this kind of methodology and start building an educational platform hosting and combining mutli-modality data.

## Data availability statement

The data analyzed in this study is subject to the following licenses/restrictions: Postmortem data. See HH074 and HH059 http://www.vhlab.umn.edu/atlas/histories/fordetails. Requests to access these datasets should be directed at: HD, halina.dobrzynski@uj.edu.pl.

## Author contributions

DP: Conceptualization, Investigation, Methodology, Writing – original draft, Writing – review & editing. PD: Conceptualization, Software, Writing – original draft, Writing – review & editing. MK: Methodology, Writing – review & editing. DD-D: Visualization, Writing – review & editing. AP: Writing – review & editing. AA: Resources, Writing – review & editing. HD: Conceptualization, Writing – review & editing. KP: Funding acquisition, Supervision, Writing – original draft, Writing – review & editing.
